# The Role of Transcranial Direct Current Stimulation (tDCS) in Tourette Syndrome: A Review and Preliminary Findings

**DOI:** 10.3390/brainsci7120161

**Published:** 2017-12-08

**Authors:** Valsamma Eapen, Richard Baker, Amelia Walter, Veena Raghupathy, Jordan J. Wehrman, Paul F. Sowman

**Affiliations:** 1 Academic Unit of Child Psychiatry South West Sydney and Ingham Institute, Liverpool Hospital, Sydney 2170, Australia; amelia.walter@unsw.edu.au; 2 School of Psychiatry, Faculty of Medicine, University of New South Wales, Sydney 2052, Australia; Richard.Baker@health.nsw.gov.au; 3 The Sydney Children’s Hospital at Randwick, Sydney 2031, Australia; 4 The Children’s Hospital at Westmead, Sydney 2145, Australia; veenaram28@gmail.com; 5 Department of Cognitive Science, Macquarie University, Sydney 2109, Australia; jordan.wehrman@mq.edu.au (J.J.W.); paul.sowman@mq.edu.au (P.F.S.); 6 Perception and Action Research Centre, Faculty of Human Sciences, Macquarie University, Sydney 2109, Australia; 7 ARC Centre of Excellence for Cognition and Its Disorders (CCD), Sydney 2109, Australia

**Keywords:** Tourette Syndrome, transcranial direct current stimulation (tDCS), treatment

## Abstract

Transcranial direct current stimulation (tDCS) is a non-invasive brain stimulation technique that is being investigated for a variety of neurological and psychiatric conditions. Preliminary evidence suggests that tDCS may be useful in the treatment of Tourette Syndrome (TS). This paper reviews the literature on the use of tDCS in commonly occurring comorbid conditions that are relevant to its proposed use in TS. We describe the protocol for a double-blind, crossover, sham-controlled trial of tDCS (Trial ID: ACTRN12615000592549, registered at www.anzctr.org.au) investigating the efficacy, feasibility, safety, and tolerability of tDCS in patients with TS aged 12 years and over. The intervention consists of cathodal tDCS positioned over the Supplementary Motor Area. Patients receive either sham tDCS for three weeks followed by six weeks of active tDCS (1.4 mA, 18 sessions over six weeks), or six weeks of active sessions followed by three weeks of sham sessions, with follow-up at three and six months. Pilot findings from two patients are presented. There was a reduction in the frequency and intensity of patients’ tics and premonitory urges, as well as evidence of improvements in inhibitory function, over the course of treatment. Larger scale studies are indicated to ascertain the maintenance of symptom improvement over time, as well as the long-term consequences of the repetitions of sessions.

## 1. Introduction

Tourette’s disorder, also called Tourette Syndrome (TS), is a childhood onset neuropsychiatric disorder, characterised by the presence of multiple motor, and one or more vocal, tics that persist for over one year [1]. Once thought to be a rare disorder, the prevalence of TS is now understood to be approximately 1% in the general population during childhood [2,3]. TS is associated with social, emotional, and academic problems, including poor social skills, low self-esteem, mood and anxiety disorders, and underperformance in school environments [4,5].

Several classes of drugs are prescribed to control tics in patients with TS, the most common being antipsychotics and alpha-2 adrenergic agonists, but these are often associated with significant side effects and are only effective in some patients [6]. Concerns about side effects are particularly pertinent for young patients. A parent survey of 740 children with TS found that 36% of parents reported that medication treatment was difficult for their child due to drug-induced side effects, and 43% of parents expressed avoidance of tic medication due to concerns about adverse effects [7].

Systematic and meta-analytic reviews have found Habit Reversal Therapy (HRT) to be an efficacious intervention for children and adults with TS [8,9]. For example, in a randomised controlled trial (RCT), Comprehensive Behavioural Intervention for Tics (CBIT), incorporating HRT, relaxation training, and trigger deterrent strategies, was found to be significantly more effective than psychoeducation and supportive therapy, with 87% of participants reporting sustained tic improvements six months post-intervention [10]. However, while there was a statistically significant decline in the Yale Global Tic Severity Scale (YGTSS) Total Tic score among both children (effect size = 0.68) and adults (effect size = 0.57) receiving CBIT when compared to the control group, roughly half of the participants receiving CBIT did not show meaningful improvement [11].

Thus, while pharmacotherapy and behavioural treatments such as CBIT are effective interventions for tics, there are still a significant number of TS patients who do not tolerate or benefit from these methods of treatment, and it is important to explore other models of intervention.

A growing body of evidence suggests that brain stimulation techniques may have a promising role in the diagnosis, monitoring, and treatment of a variety of neurological and psychiatric conditions, including TS. For example, Transcranial Magnetic Stimulation (TMS), a non-invasive technique, may correct abnormal cortical excitability activity in patients with TS through the repetitive generation of a brief, powerful magnetic field by a coil positioned over the scalp that induces an electric current in the brain [12]. Previous research indicates that TMS is safe and well-tolerated in children and adolescents, with only mild and temporary side effects reported, including headache and sleepiness [12,13]. TMS has also been shown to be effective in the treatment of TS among children and adolescents aged 7–16 years, with significant improvements in clinical symptoms lasting up to six months [12]. However, TMS requires extensive training to administer and is non-portable. Deep Brain Stimulation (DBS) is also being increasingly used to treat refractory cases [14]. DBS, however, has obvious limitations being significantly invasive. Primary benefits of tDCS, another non-invasive brain stimulation technique, are its affordability, portability, and ease of use, with the potential for self-administration by patients at home. Despite such promising attributes, there is very little evidence to date to support the use of tDCS in the treatment of TS. This paper provides a review of the literature relevant to the use of tDCS in TS, and preliminary findings from a pilot study aimed to evaluate the use of tDCS to treat TS by targeting an important locus of inhibitory control in the cortex, proposed as the primary deficit in TS [15,16,17].

### 1.1. Background to tDCS

tDCS is a form of neuromodulation that uses a constant, low current, delivered to the cortical brain area of interest via electrodes positioned on the scalp. The current modulates the neuronal excitability by altering the resting membrane potential of the stimulated neurons [18]. Anodal stimulation is used to increase cortical excitability in the stimulated area, while cathodal stimulation is used to inhibit neuronal excitability [19]. Current delivery has the capacity to induce neuromodulatory changes in cortical activity, including prolonged changes in neuronal excitability beyond the period of direct stimulation [20,21]. Sustained changes in neuronal excitability are thought to be driven by a cascade of events at the molecular and cellular level involving glutaminergic, dopaminergic, serotonergic, and GABAergic activity modulation and synaptic plasticity [22,23].

tDCS is more affordable, flexible, and simple to operate than other neuromodulation techniques and is becoming one of the most investigated of the non-invasive brain stimulation modalities [23]. A factor influencing attrition rates for neurostimulation treatments is the need for consecutive dosing regimens. Supporting the flexibility in scheduling of tDCS treatment regimes, a study assessing the impact on efficacy of non-continuous treatment sessions found that scheduling up to two treatment-free days during a ten-day acute treatment regime did not modify the clinical efficacy of tDCS treatment for depression [24]. The potential for increased flexibility in treatment regime and the capacity for tDCS to be undertaken at home is likely to afford greater access to treatment and superior retention rates when compared to solely clinic-based neurostimulation interventions such as TMS.

The safety and tolerability of tDCS has been well established in adults, with the limited adverse side effects being uncommon, mild, and short-lived [25]. There is a building evidence base to show that tDCS is safe and well-tolerated among children and adolescents with a range of neurological and psychiatric disorders [18]. In a literature review that included 191 child subjects who underwent tDCS, no children experienced any major side effects. Transient side effects reported include scalp redness, tingling, itching, and scalp discomfort. Side effects were experienced for no longer than two hours and in less than 12 percent of children and adolescents [18]. Nevertheless, studies with longitudinal assessment are warranted in children, and further data on the tolerability within specific dosage parameters will be useful in the development of safety guidelines for the use of tDCS in this age cohort.

### 1.2. Indications for tDCS Reported in the Literature for Commonly Occurring Comorbid Conditions with TS

Research has demonstrated the efficacy of tDCS for the treatment of a range of psychiatric conditions that often occur comorbidly with TS, including Major Depressive Disorder (MDD), Obsessive Compulsive Disorder (OCD), and Attention Deficit Hyperactivity Disorder (ADHD) [26]. Novel research has described its utilisation in the treatment of movement disorders [27], Alzheimer’s disease [28], addiction [29], tinnitus [30], eating disorders [31], and psychosis [32,33]. Research into its capacity to improve the cognitive deficits associated with various disorders has also begun, with encouraging early results [34]. The following is a brief introduction to the indications for tDCS for the treatment of conditions that are commonly comorbid with TS that may have related underlying deficits. Thorough reviews of the wider indications for tDCS are available elsewhere (see, for example [35,36,37]).

**MDD:** Depression has been the most extensively studied indication for tDCS in the treatment of psychiatric disorders. The mechanism of action of tDCS for the treatment of depression is speculated to involve enhancement of neural activation in the left dorsolateral prefrontal cortex (DLPFC) via anodal stimulation and/or reducing neural activity on the right DLPFC via cathodal stimulation [38]. Several meta-analyses have concluded that tDCS produces robust and clinically meaningful effects in the treatment of depression [39,40,41]. Among patients with depression, tDCS has also been found to result in improved attention, concentration, and psychomotor speed [42]. A recent meta-analysis of individual patient data concluded that tDCS treatment for acute depressive episodes produced comparable positive effect sizes to those reported for antidepressant drug therapy in primary care and for TMS [40]. Further, a recently published evidence-based guideline for the therapeutic use of tDCS supported its use in the treatment of MDD using anodal tDCS over the left DLPFC in patients with and without drug-treatment-resistant depression [37].

The longer-term efficacy of tDCS in treating major depression has been examined in one study that demonstrated a sustained anti-depressant effect without maintenance therapy at three-month follow up [43]. Additionally, factors influencing relapse rates post tDCS were investigated in a study that demonstrated increased relapse rates in patient groups with a higher level of treatment resistance prior to commencing tDCS [44].

**OCD:** tDCS has shown promising results in the treatment of OCD [45]. A case series observed significant clinical improvement utilising tDCS as an adjunctive therapy with routine selective serotonin reuptake inhibitor (SSRI) treatment in SSRI-resistant OCD [46]. A preliminary study demonstrated cathodal but not anodal tDCS applied over the pre-Supplementary Motor Area (SMA) significantly improved OCD symptoms [47]. A recent case study of twice-daily tDCS over 10 days also demonstrated significantly decreased OCD symptoms, as well as reduced symptoms of depression and anxiety [48].

**ADHD:** Contemporary interest has been reported in the literature regarding the use of tDCS in the treatment of ADHD in both children and adults [49,50]. A recent exploratory study demonstrated that anodal tDCS treatment targeting the prefrontal cortex significantly reduced the clinical symptoms of inattention and impulsivity in adolescents with ADHD [51]. Supporting the potential use of tDCS in the treatment of ADHD has been the reported post-tDCS improvement in behavioral inhibition, attention, and memory in healthy clinical populations [34,50,52,53]. The use of tDCS to enhance psychological interventions such as cognitive training has also shown promising results. Further research into the efficacy of tDCS as a combination treatment within other psychological treatment frameworks is warranted [54].

### 1.3. Treatment Factors Affecting tDCS Efficacy

While early results are promising for the use of tDCS in the treatment of various neurological and psychiatric disorders, predictors of individual response and optimisation of stimulation parameters including electrode placement, electrode size, current dosage, and frequency of treatments are yet to be established [40,55]. Consistency of reporting and use of specific parameters in future research will be important in assessing the relationship between treatment parameters and efficacy.

Electrode positioning appears to be critical despite the tDCS electrical fields being relatively non-focal [56,57]. The impact of how neural stimulation in a specific cortical area affects adjacent areas remains unclear, and the potential to simultaneously increase and decrease activation in separate cortical regions could be explored [58]. Optimising the efficiency of tDCS current delivery via electrode placement and specific electrode configurations with the use of computational models has been demonstrated in the literature and may be another avenue for further optimisation of efficacy [59]. This provides a potential avenue for research into the improvement of tDCS treatment efficacy for various neuropsychiatric indications via more selective stimulation targets.

Preliminary research into the potential effects of pharmacological agent exposure and augmentation in relation to predictors of response to tDCS has generated interesting findings. Enhancement of anodal tDCS effects on cortical excitability has been demonstrated with pharmacological augmentation by SSRIs [60], amphetamines (a noradrenergic transporter and competitive inhibitor of dopamine transporters) [61], and D-cycloserine (an NMDA agonist) [62]. Similarly, the effect of pharmacological agents on cathodal tDCS has been explored. Cathodal tDCS effects have demonstrated enhancement with L dopa (a dopamine precursor) [63], and rivastigmine (a cholinesterase inhibitor) has demonstrated a stabilisation of cathodal tDCS effects [64]. In clinical samples, early findings from a naturalistic study suggested the efficacy of tDCS for the treatment of MDD may be reduced in patients who have had prior benzodiazepine treatment and improved with those who had prior treatment with dual reuptake inhibitors, indicating prospects for further research [65]. A recent study examining clinical predictors of response to tDCS among patients with treatment-resistant depression showed that cognitive disturbance and psychomotor retardation were predictive factors for a reduction in depressive symptoms following tDCS [66].

### 1.4. Proposed Mechanism of Action of tDCS for the Treatment of TS

Neuroimaging has implicated the SMA as a locus of dysfunction in TS, with activity in the SMA being positively correlated with tic severity [67,68]. However, the role of SMA in TS could be either generative, i.e., SMA over-activation represents the generation of urge [69], or conversely, given the key role that SMA is thought to play in the network for inhibitory control [70], it could represent an attempt at tic suppression. Significant TMS work shows that suppressing excitability in the SMA leads to a reduction of tics [71,72,73,74], favouring the explanation that over-activation in the SMA may contribute to the genesis of TS. Furthermore, it has been demonstrated recently that cathodal (inhibitory) tDCS over the SMA can prevent the behavioural expression of action impulses [75]. It is therefore expected that the administration of cathodal tDCS to the SMA among patients with TS would result in a decrease in premonitory urges and a reduction in the frequency and severity of patients’ tics.

### 1.5. Previous Research on the Use of tDCS for Treatment of TS

Excepting a promising report of two adult cases with TS who received cathodal tDCS over the motor areas of the cerebral cortex [76], and a case report on a 16 year-old investigating the effect of cathodal tDCS over the pre-SMA in a patient with TS [77], no blinded study to date has investigated tDCS as a therapeutic intervention for TS. This is despite extensive previous research demonstrating its safety and tolerability across age ranges in other neurological and common comorbid psychiatric disorders [18].

## 2. Description of a Study Protocol for a Double-Blind, Crossover, Sham-Controlled Trial of tDCS

In this context, we sought to undertake a clinical pilot of a double-blind, crossover, sham-controlled trial of tDCS, examining the feasibility, safety, tolerability, and preliminary efficacy of tDCS for treatment of TS in individuals aged over 12 years (Trial ID: ACTRN12615000592549, registered at www.anzctr.org.au). This study will provide preliminary data on efficacy outcomes that will be useful in informing a subsequent RCT. The study is planned as the first-ever extensive clinical trial of tDCS to the SMA as a therapy for TS. The protocol is described in the remainder of this paper, and data presented from two adult participants on whom the protocol was piloted.

### 2.1. Hypotheses

We hypothesise that administration of tDCS, by electrically stimulating a cortical area important for the generation and control of movements, namely the SMA, will result in improved inhibitory control and a decrease in the frequency and severity of participants’ tics. We also propose that the ability to rapidly suppress prepotent action, as measured by a modified go no-go task, will be negatively correlated with the occurrence of tics among patients with TS.

### 2.2. Methods

**Study design:** Participants will receive a course of tDCS, three times per week for six weeks (a total of 18 tDCS sessions). To allow for contingencies, participants will be required to complete a minimum of two and a maximum of three sessions in any one week, and to complete the total 18 sessions. Participants will be tested in a crossover design with double-blind random allocation, whereby half the participants will receive sham sessions for three weeks followed by six weeks of tDCS, while the other half will receive six weeks of active sessions followed by three weeks of sham sessions. Symptom severity measures will be administered at several time points (baseline, Week 3, Week 6, Week 9, 3 months, and 6 months).

**Sampling:** We aim to recruit eight participants for the initial clinical study, not including the two adult participants that the study protocol has been piloted on. As this is not a definitive RCT, sample size is not based on formal power calculations based on effect sizes. The proposed sample size is comparable to that used in several trials of non-invasive brain stimulation procedures among children and adolescents [18].

**Inclusion criteria:**

1. Participant aged over 12 years (written consent will be provided by parent/guardian for those aged 12 to 18 years).

2. Participant meets criteria for a primary diagnosis of DSM-5 Tourette’s Disorder: presence of multiple motor and one or more vocal tics that have persisted for more than one year with onset before 18 years of age [1].

3. Participants taking psychotropic medications to manage tics will be included as long as the dose had been stable for at least six weeks prior to participation, with no planned changes during the course of the study.

**Exclusion criteria:**

1. Unacceptable risk factors for unsafe administration or side effects of tDCS (implanted cranial devices, previous head or brain injury, skin lesions on scalp at proposed electrode sites, epilepsy or history of seizures, drug abuse).

2. Participants not fluent in English will be excluded from the trial for safety reasons.

3. Participants who have a primary psychiatric or medical diagnosis apart from TS. While participants with comorbidities commonly associated with a primary diagnosis of TS (such as OCD and ADHD) will be included, those with any major psychiatric conditions such as psychosis or Bipolar Disorder will be excluded.

4. Pregnancy.

**Recruitment:** Participants will be recruited to the study through an advertisement via the Tourette Syndrome Association of Australia’s website.

**tDCS administration:** tDCS will be administered via sponge electrodes (stimulating electrode 25 cm^2^) soaked in saline and held in place by a neoprene cap, with the cathode positioned over the anterior portion of the SMA (4 cm in front of the vertex/Cz) and the return electrode positioned over the right deltoid. The current intensity will be given at up to 1.4 mA (milliamps) for a duration of 20 min per session. The first two treatment sessions will be undertaken at the research centre and the remaining treatments will be conducted in patients’ homes using a portable unit. Settings on the unit will be pre-programmed and unable to be changed by the participants. All sessions conducted at participants’ homes will be supervised remotely by the study’s medical practitioner via audio-visual link. Parental supervision during treatment will be required for all participants aged under 18 years.

**Outcome measures:** Outcome measures will be administered at baseline, week three, week six, week nine, three months, and six months, with the exception of inhibitory function, which will not be assessed at three-month follow-up.

*Tic frequency and severity:* Tic frequency and severity will be assessed via the YGTSS [3]. A brief version of the *National Hospital Interview Schedule for the assessment of Gilles de la Tourette syndrome* (NHIS), the Tic Screening Questionnaire, will be administered at baseline to objectively evaluate the presence and severity of overall tics and related behaviours as well as comorbidities.

*The Parent Tic Questionnaire* (PTQ) and the parallel *Adult Tic Questionnaire* (ATQ) will be used as measures of tic severity during the previous week [78]. Participants (either the patient or his/her parents) will be asked to indicate the presence or absence of 14 motor and 14 vocal tics. Participants will then be asked to rate each endorsed tic on a scale of 1 to 4 in both frequency and intensity, such that scores for each tic range from 0 (tic not present, i.e., if participants indicate absence of the tic) to 8 (maximum frequency and intensity). The sum of all 28 individual tic scores yields a total score (maximum score 224), with higher scores indicating greater tic severity. The measure shows good internal consistency, temporal stability, and convergent and discriminant validity [78].

*The Premonitory Urge for Tics Scale* (PUTS) will also be utilised as a self-report scale designed to measure the subjective intensity and character of premonitory urges in TS [79]. The PUTS includes ten qualitative items rated on a scale ranging from 1 (not at all true) to 4 (very much true), generating a total score between 10 and 40 that indicates both the frequency and intensity of premonitory urges. The PUTS has good internal consistency, temporal stability, and convergent validity when used with children aged over 10 years [79].

*Inhibitory function:* Alterations in the ability to appropriately inhibit prepotent motor actions have been linked to TS [80,81]. The Go No-Go task (GNG; [82]) is a psychophysical measure designed to assess ability to inhibit inappropriate actions. In this task, participants are instructed to make rapid responses to (usually) visually presented “Go” signals and to withhold that response when the signal is a “No-Go”. “Go” signals are typically more frequent than “no-go” signals so that the tendency to respond is dominant and becomes the prepotent state. The primary measure that indexes the potency of inhibitory function is the rate of false alarms following the presentation of the no-go signal. In this study, we will use the modification demonstrated by Los [83] that combines the explicit inhibitory measures of the GNG task with implicit inhibitory control measures embedded in the variable foreperiod paradigm. The variable foreperiod design presents participants with response imperatives at one of two or more intervals, requiring withholding of the response throughout the duration of the foreperiod until the imperative indicates that it is appropriate to activate the response. This novel approach presents a methodological advancement in the study of inhibitory control in TS, as a limitation of current measures of inhibitory control is the ability of the patient to make changes to their response tendencies in order to compensate for inhibitory deficits [84,85]. The GNG task, as a measure of the ability to inhibit inappropriate responses, has good reliability for commission errors [86], which, when combined with a strong factor structure [87], indicates a strong internal structure [88]. Further, the GNG task demonstrates strong content validity [89], precisely measuring the ability of the participant to withhold a response. Importantly, in respect to the current study, the GNG has been shown to be sensitive to interventions [90] and associated with clinical outcomes [91].

**Assessment of side effects:** At the end of each tDCS session, a medical practitioner on the research team will screen for the presence of any of the following side effects: itchiness, dizziness, light headedness, fatigue, blurred vision, headache, or nausea. If of any of these are answered positively, the duration of the symptom/s will be monitored and, if these do not dissipate within an hour, which is typical of tDCS, the participant will be referred for assessment by an on-call medical practitioner.

**Pilot findings and discussion**: The study protocol, up to and including the three-month follow-up, has been piloted on two adult participants, both male. These participants will not be included in the actual trial, and thus publication of results does not compromise blinding or constitute double reporting. Using random allocation, both pilot participants received six weeks of active tDCS treatment first, followed by three weeks of sham stimulation. They remained blind to this. The sessions were well tolerated and no side effects were reported by either of the two participants. YGTSS data was not collected for these participants, but will be for all future participants.

*ATQ scores:* As shown in Table 1, there was a reduction in the frequency and severity of both pilot participants’ tics (as rated using the ATQ) over the course of treatment. Participant 1’s ATQ score further decreased between the end of treatment and the three-month follow-up. However, there was an increase in Participant 2’s ATQ score over this same period such that his ATQ score at three-month follow-up indicated greater tic severity and frequency than at baseline. Given the preliminary nature of the data, it is not possible to determine whether improvements that had been made over the course of treatment were not maintained due to treatment inefficacy, or whether additional stressors that were present at the time of the three-month follow-up may have adversely affected this participant’s tics.

*PUTS scores:* As shown in Table 2, there was a reduction in the frequency and intensity of both pilot participants’ premonitory urges (as rated using the PUTS) over the course of treatment. Following treatment, both participants had scores (22 and 24 for Participant 1 and 2, respectively) that fell in the medium intensity range for premonitory urges, compared to pre-treatment scores that indicated high intensity (25) and extremely high intensity with probable severe impairment (34) for Participants 1 and 2, respectively. These improvements were largely maintained for both participants between the end of treatment and the three-month follow-up.

**Figure 1:**
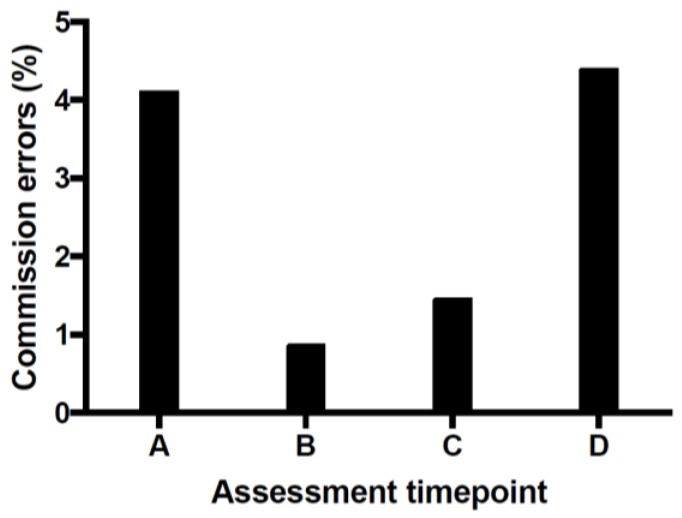
Mean commission errors as a percentage of total no-go trials (*n* = 2). The percentage of commission errors reduced during the active treatment phase and then rebounded post-treatment. Post-treatment is after the three weeks of sham stimulation. A: Pre-treatment; B: After 3 weeks of active transcranial direct current stimulation (tDCS); C: After 6 weeks of active tDCS; D: Post-treatment (6 weeks active + 3 weeks sham).

*Inhibitory function:* The main dependent variable on the go-no go task is the proportion of commission errors on the no-go trials. Figure 1 shows the mean percentage of commission errors across the two participants as a function of treatment status. Both participants displayed a surprisingly low rate of commission errors at baseline, likely in part due to the relatively high proportion of no-go trials we used. However, on average, participants exhibited a reduction in the rate of commission errors during the active phase of tDCS that seemed to rebound once the active treatment ceased. Note that inhibitory function is not assessed at three-month follow-up.

## 3. Conclusions

Available evidence from the literature and our pilot findings suggest that tDCS is safe and well tolerated. Data from our two pilot participants revealed clinical improvement, suggesting a potential role for tDCS in the treatment of TS. However, the mechanism of action is not clear. At this early stage, there does seem to be a reduction in the number of commission errors committed in the go no-go task between the active vs. sham phase of tDCS. However, given that this reduction did not persist beyond the treatment phase, rebounding to baseline once tDCS treatment ceased, it seems less likely that the mechanism for the enhancement in voluntary inhibition is directly linked to the clinical improvement. *Future studies involving* a larger number of participants are indicated, with long term follow-up to ascertain the maintenance of symptom improvement over time.

## Figures and Tables

**Table 1:** Adult Tic Questionnaire (ATQ) scores.

	Pre-Treatment	After 3 Weeks of Active tDCS	After 6 Weeks of Active tDCS	Post-Treatment (6 Weeks Active + 3 Weeks Sham)	3 Months Post-Treatment
Participant 1	83	66	54	54	47
Participant 2	34	29	27	18	39

**Table 2:** Premonitory Urge for Tics Scale (PUTS) scores.

	Pre-Treatment	After 3 Weeks of Active tDCS	After 6 Weeks of Active tDCS	Post-Treatment (6 Weeks Active + 3 Weeks Sham)	3 Months Post-Treatment
Participant 1	25	22	23	22	22
Participant 2	34	29	27	24	25
